# Study on the molecular mechanism of matrine in improving rheumatoid arthritis by targeting the NAV2-Wnt3a/β-catenin axis to coordinately regulate the inflammatory-osteolytic loop

**DOI:** 10.3389/fimmu.2025.1646918

**Published:** 2025-08-27

**Authors:** Qianyu Guo, Meie Liang, Zhen Li, Jie Li, Ke Xu, Liyun Zhang

**Affiliations:** ^1^ Third Hospital of Shanxi Medical University, Shanxi Bethune Hospital, Shanxi Academy of Medical Sciences, Tongji Shanxi Hospital, Taiyuan, China; ^2^ Department of Rheumatology, Taiyuan, China; ^3^ Stem cell Translational Laboratory, Taiyuan, China

**Keywords:** matrine, rheumatoid arthritis, collagen-induced arthritis, NAV2-Wnt3a/β-catenin axis, inflammatory-osteolytic loop

## Abstract

**Background:**

The occurrence and development of rheumatoid arthritis (RA) are closely related to bone erosion caused by the abnormal activation of the Wnt3a/β-catenin signaling pathway. However, it remains unclear how natural products target and regulate this pathway at the molecular level. This study focuses on the role of the novel neuronal guidance protein NAV2 in RA and systematically analyzes the immunoregulatory mechanism of matrine (MAT).

**Method:**

A Wistar rat model of type II collagen-induced arthritis (CIA) was established by co-induction with bovine type II-collagen and Freund’s adjuvant. Twenty-four rats were randomly divided into four groups in total: a normal control group (NOR), a model group (CIA), a methotrexate treatment group (MTX), and a matrine treatment group (MAT). Intragastric administration was carried out over four weeks.Ankle bone mineral density (BMD), trabecular thickness (tb.th) and other related indicators were detected. The dynamic balance of serum inflammatory factors was measured via enzyme-linked immunosorbent assay (ELISA). The destruction of articular bone was gauged utilizing three-dimensional reconstruction with Micro-CT. Hematoxylin and eosin (HE) staining, along with O-fast green staining, were employed to appraise synovial inflammation and the degree of cartilage damage in the joints. qRT-PCR and Western blotting were utilized to detect the mRNA and protein expression levels of NAV2, Wnt3a, and β-catenin in rat joints.

**Result:**

MAT remarkably decreased the arthritis index in CIA rats (p<0.01), and effectively ameliorated articular bone erosion and synovial inflammatory infiltration. MAT systematically reduced the levels of pro-inflammatory cytokines, while increasing the levels of anti-inflammatory cytokines. No significant statistical differences were observed in liver and kidney function between the MAT group and NOR group (p>0.05). MAT significantly inhibited the mRNA and protein expressions of NAV2, Wnt3a, and β-catenin in joints (all p<0.05). Following intervention with MAT, there was a significant decrease in the positive areas of these three molecules (p<0.01).

**Conclusion:**

Through the targeted inhibition of the NAV2-Wnt3a/β-catenin signal transduction pathway, MAT exerts a dual-regulatory effect to restore the equilibrium of the inflammatory cytokine network. Moreover, MAT demonstrates no hepatotoxicity or nephrotoxicity, thereby providing a novel candidate molecule and a mechanistic target for the intervention of autoimmune diseases using natural drugs.

## 1 Introduction

Rheumatoid arthritis (RA) is a systemic autoimmune disease clinically characterized by synovial inflammation, bone erosion, and joint deformity. Epidemiological studies have shown that the global prevalence of RA is between 0.5% and 1% (1, 2). From a pathophysiological perspective, the progression of RA is driven by the abnormal activation of fibroblast-like synoviocytes (FLS), the occurrence of a pro-inflammatory cytokine storm, and the establishment of a bone-erosive microenvironment. These pathological processes ultimately lead to joint deformity and functional impairment (3). Currently, the mainstay of clinical treatment for RA includes Janus kinase (JAK) inhibitors, biological agents (e.g., TNF-α inhibitors), and traditional disease-modifying antirheumatic drugs (DMARDs). Nevertheless, the long-term administration of these medications is often associated with various adverse reactions, such as increased susceptibility to infections, metabolic disturbances, and the development of drug resistance (4). Thus, there is an urgent need to develop innovative treatment strategies that can strike a balance between therapeutic efficacy and safety. Accumulating evidence from numerous investigations has demonstrated that natural medicines exert anti-arthritic effects through multiple mechanisms. These mechanisms encompass immunomodulation, antioxidant stress, inhibition of angiogenesis, mitigation of bone destruction, and downregulation of inflammatory factor expression, among others (5, 6). Extracted from Sophora flavescens, a herb in traditional Chinese medicine, MAT is a quinolizidine alkaloid characterized by the molecular formula C_15_H_24_N_2_O and a molecular weight of 248.36 g/mol. With the continuous advancement of modern pharmacological research, the medicinal potential of MAT has been increasingly explored. It demonstrates multiple pharmacological properties, including anti-inflammatory, immunomodulatory, and organ-protective actions (7–10). Modern pharmacological studies indicate that MAT exerts its effects by targeting key signaling pathways such as PI3K/AKT/mTOR, NF-κB, JAK/STAT, and MAPK, which play crucial roles in regulating immune responses. By regulating the equilibrium of inflammatory cytokines and immune cells, MAT can impede the progression of RA (11). However, the underlying mechanisms of its action in RA remain unclear. Previous studies have shown that MAT effectively reduces joint swelling and attenuates bone damage in type II collagen-induced arthritis (CIA) mice (12). Additionally, MAT has been shown to downregulate the Th17/Treg cell ratio, thereby improving the immune microenvironment within the joints (13). However, the influence of MAT on the crosstalk between synovium and cartilage tissues, as well as its effects on the key signaling networks implicated in the pathogenesis of RA, remain largely undefined. The Wnt3a/β-catenin signaling pathway plays a dual role in the pathological progression of RA. Abnormal activation of this pathway not only drives the invasive proliferation of fibroblast-like synoviocytes (FLS) (14), but also intensifies bone destruction by disrupting the balance of the RANKL/OPG signaling axis (15). Accumulating evidence from previous investigations has indicated that neuronal navigator protein 2 (NAV2), serving as an upstream regulator of the Wnt3a pathway, It also promotes the release of inflammatory factors through the nuclear translocation of β-catenin in fibroblast-like synoviocytes (FLS). This process suggests that NAV2 may act as a potential determinant for the perpetuation of chronic inflammation in RA (14). Notwithstanding the pivotal role of the NAV2-Wnt3a/β-catenin axis within the regulatory network of RA. However, the precise molecular mechanisms by which MAT targets and modulates this signaling cascade are still poorly understood. Hence, the present study aims to elucidate the coordinated regulation of the inflammatory-osteolytic loop by the NAV2-Wnt3a/β-catenin axis. It will conduct a comprehensive analysis of the immunoregulatory mechanisms underlying MAT’s therapeutic effects in RA treatment. This thereby establishes an experimental basis for the targeted utilization of natural drugs in the management of RA.

## 2 Experimental materials and methods

### 2.1 Grouping of experimental animals and model construction

A total of twenty-four specific pathogen-free (SPF)-grade healthy female Wistar rats (6–8 weeks old, weighing 160 ± 10 g) were selected for this study. The rats were procured from SpePharm (Beijing) Bio. Co., Ltd., China (License no. SCXK (Jing) 2019-0010) and housed at the Experimental Animal Center of Shanxi Bethune Hospital (Taiyuan, Shanxi, China). Using the random number table method, the rats were randomly assigned to four groups (six rats per group): healthy control group (NOR), collagen-induced arthritis model group (CIA), CIA group treated with intragastric methotrexate (MTX, positive drug), and CIA group treated with intragastric MAT. The CIA models were established in all groups except NOR. The protocol for CIA model establishment was as follows: Following a one-week adaptive feeding period, primary immunization was initiated. Rats were intradermally injected at three dorsal sites and two sites at the tail base with an emulsified mixture (v:v 1:1) of Freund’s complete adjuvant (Lot. No. F5881, Sigma-Aldrich Pty Ltd, St. Louis, MO, USA) and bovine type II collagen (Catalog No. 20022, Chondrex, Inc., Redmond, WA, USA), at a dosage of 0.5 mL per rat. One week later, booster immunization was performed, during which rats were intraperitoneally injected with an emulsified mixture (v:v 1:1) of Freund’s incomplete adjuvant (Lot. No. F5506, Sigma-Aldrich Pty Ltd, St. Louis, MO, USA) and bovine type II collagen at a dosage of 0.3 mL per rat. Arthritis scores and joint swelling degrees of rats in each group were recorded weekly. Rat paw edema was measured using the ZH-ZZY Type Toe Volume Measuring Instrument (Anhui Zhenghua Bio-Instrument Equipment Co., Ltd., Anhui, China). The arthritis scoring criteria were defined as follows: 0 points, normal; 1 point, mild joint redness, and swelling; 2 points, moderate joint redness and swelling; 3 points, obvious paw redness and swelling; 4 points, obvious swelling of the entire foot, joint deformation, and impaired mobility. A total score of the toes of the four limbs > 8 points (out of a maximum of 16 points) was considered indicative of successful model establishment. All experimental rats were provided with Maintenance feed for rats and mice (Catalog No. SPF-F02-001, SpePharm Bio. Co., Ltd., Beijing, China) as their daily diet. The photoperiod was set to a 12-hour light/12-hour dark cycle. The environmental conditions were strictly controlled, with the temperature maintained at (23 ± 2)°C and the humidity at (50 ± 15)%. Throughout the experiment, the rats had free access to food and water ad libitum. The animal experimental protocol was reviewed and approved by the Experimental Animal Ethics Committee of Shanxi University of Chinese Medicine (Approval No. AWE202411433), ensuring adherence to ethical standards for animal research.

### 2.2 Administration of drugs and collection of samples

Eighteen successfully established CIA model rats were randomly allocated into three groups (n=6) according to body weight: the collagen-induced arthritis model (CIA) group, which received intragastric administration of 2.0 mL/d normal saline; the methotrexate (MTX) treatment group, administered intragastrically with 0.9mg/kg.d MTX (Catalog No.197221004, Shanghai Sine Pharmaceutical Laboratories Co., Ltd., Shanghai, China), with the dosage calculated as the equivalent dose according to the body surface area ratio between humans and rats; and the MAT treatment group, receiving 150 mg·kg·d MAT (Catalog No. T92781, Shanghai Yuanye Bio-Technology Co., Ltd., Shanghai, China). The dosage was also calculated based on the human-rat body surface area ratio. All groups received continuous intragastric administration for four weeks. Following the final administration, the rats in each group were fasted overnight for 12 hours. Subsequently, all experimental animals were anesthetized with isoflurane gas, and tissue samples were collected to minimize pain, distress, and mortality. Blood was collected from the abdominal aorta and allowed to clot at room temperature for 30 minutes, followed by centrifugation at 3,000 r·min for 10 minutes. The separated serum was stored at -80 °C. Half of the spleen, kidney, and liver tissues, along with the left ankle joint, were fixed in 4% paraformaldehyde. The other half of these tissues and the right ankle joint were stored at -80 °C for subsequent analysis.

### 2.3 Histopathological examination of the spleen and joint tissues from rats in different experimental groups

The spleens and joints of the rats were immersed in 4% paraformaldehyde for fixation, which lasted over 24 hours. Subsequently, the joints were subjected to a 5-week decalcification treatment in a 10% EDTA decalcification solution. Following decalcification, the tissues underwent a sequential processing protocol: gradient ethanol dehydration, xylene clearing, paraffin infiltration and embedding, sectioning into 5-μm-thick slices, mounting on slides using 37 °C warm water, baking at 60 °C, xylene dewaxing, rehydration through gradient ethanol series, and finally hematoxylin-eosin (HE) staining using a commercial kit (Catalog No. DH0006, Beijing Leagene Biotechnology Co., Ltd., Beijing, China). For the Modified Safranin O-Fast Green Cartilage Staining, the staining was conducted following the instructions of the staining kit (Catalog No. G1371, Solarbio Sci. & Tech. Co., Ltd., Beijing, China). Initially, routine dewaxing and rehydration procedures were performed. The samples were then stained with Weigert hematoxylin for 5 minutes, rinsed with water, treated with an acidic differentiating solution for 15 seconds, and rinsed with distilled water for 10 minutes. Next, they were stained with O-Fast green staining solution for 5 minutes, rinsed with a weak acid solution for 15 seconds, and stained with safranin staining solution for 5 minutes. Afterward, the samples were dehydrated with absolute ethanol for 1 minute, cleared with xylene, and sealed with neutral gum. Histopathological changes in the spleen and joint tissues of rats from different experimental groups were observed under an optical microscope (RM2665, Leica Microsystems GmbH, Wetzlar, Germany).

### 2.4 Micro-CT scanning of the rats’ ankle joints

The fixed hind limbs of the rats were retrieved, and the excess muscles were carefully dissected and removed for subsequent processing. A high-resolution *in vivo* micro-computed tomography (micro-CT) system (Skyscan 1276, Bruker Scientific Instruments Hong Kong Co. Ltd., Hong Kong, China) was used to scan the joints of rats in each experimental group. Three-dimensional reconstruction of the scanned images was performed using CTvox software (Bruker), and the CTan analysis software (Bruker) was employed to quantitatively analyze the BMD, the ankle joints’ BV/TV, Tb.Th, and Tb.Sp.

### 2.5 Detection of serum inflammatory factor levels in rats across different experimental groups

ELISA kits (Catalog No. ml002859, ml003057, ml064292, ml037371, ml107039, ml037365; Enzyme-linked Biotech. Co., Ltd., Shanghai, China) were utilized to detect the levels of serum inflammatory factors, includingTNF-α, IL-6, IL-1β, IL-13, IL-10, and IL-17A, in rats of each experimental group. In strict accordance with the kit instructions, standard wells, blank wells, and sample wells (in triplicate) were set up. The experimental procedures, encompassing dilution, enzyme addition, incubation, washing, color development, and reaction termination, were meticulously performed in sequence. Using a fully automatic microplate reader (ELX808, BioTek Instruments, Inc., Winooski, VT, USA), the absorbance of each well was measured at 450 nm. The concentrations of inflammatory factors in the samples were then calculated using the standard curve.

### 2.6 Evaluation of hepatic and renal function parameters alongside histopathological analysis of rats across experimental groups

The serum concentrations of ALT, AST, AKP, CRE, UREA, and UA were quantified using a fully automated biochemical analyzer (BS-180, Mindray Bio-Medical Electronics Co., Ltd., Shenzhen, China). Liver and renal tissues were harvested from rats across experimental treatment groups. Collected tissues were processed for paraffin embedding: trimmed paraffin blocks underwent gradient ethanol dehydration, xylene clearing, re-immersion in molten paraffin, and subsequent embedding. Tissues were sectioned into 5-μm-thick slices using a microtome. Following routine dewaxing and rehydration, sections were stained with hematoxylin-eosin (HE) and coverslipped with neutral gum. Pathological changes in tissues were then visualized under an optical microscope (RM2665, Leica Microsystems GmbH, Wetzlar, Germany).

### 2.7 Detection of the mRNA levels of the core genes in the NAV2-Wnt3a/β-catenin pathway in the joints and joint tissues of rats

The frozen joint tissues of rats from each experimental group were retrieved, accurately weighed, and then total RNA was isolated from joint tissues using the Total RNA Extraction Kit (Catalog No. G1492, Solarbio), following the manufacturer’s protocol. The concentration and purity of the extracted RNA were assessed using a UV-Vis spectrophotometer (UV-1800PC, Shanghai Mapada Instruments Co., Ltd., Shanghai, China). Qualified RNA samples meeting predefined quality criteria were stored at -80°C for subsequent use. Reverse transcription was performed using the RT mix with DNase (All-in-One) kit, in strict accordance with the kit’s operational manual. A 20 μL reaction system was constructed as follows: For reverse transcription, 4.0 μL of UEIris 5×RT All-in-One Mix and 1.0 μL of DNase were added to 0.05–1 μg of RNA template, with the total reaction volume adjusted to 20 μL using RNase-free water. The mixture was incubated at 37°C for 2 min, 55°C for 15 min, and 85°C for 5 min, followed by storage at -20 °C until further use. For quantitative real-time polymerase chain reaction (qRT-PCR), a 20 μL reaction system was prepared according to the TransStart^®^ Top Green qPCR SuperMix kit (Catalog No. S2024L, US Everbright) protocol, consisting of 10 μL of 2×Universal SYBR Green qPCR Supermix, 0.5 μL of each forward and reverse primer (10 μmol/L), and 2 μL of Template DNA were added, and the volume was made up to 20 μL with ddH_2_O. The reaction mixtures were subjected to amplification in a real-time fluorescence quantitative PCR system (LightCycler 96, Roche Diagnostics, Basel, Switzerland) with technical triplicates for each sample. Thermal cycling conditions comprised: initial denaturation at 95°C for 30 sec (1 cycle); 35 cycles of denaturation at 95°C for 15 sec; 40 cycles of combined annealing/extension at 60°C for 35 sec; and a dissociation curve analysis (1 cycle: 97°C for 30 sec, 65°C for 1 min, 97°C for 10 sec). Primers (**Table 1**) were commercially synthesized by TsingKe Biotechnology Co., Ltd. (Beijing, China). Relative quantification of target gene mRNA levels across experimental groups was performed using the 2^^-^Ct^ method.

**Table 1:** Primer sequence information.

Genes	Accession No	Primer Sequences (5'→3')	Product length/bp
NAV2	NM 138529.1	F:ACTGGTCCTCAACCCTGCTA R:GTCGGTCACATCTTGCTGGA	249
Wnt3a	NM_001414349.1	F:ACGATGGCTCCTCTCGGATA R:ACCACCAAATCGGGTAGCTG	76
β-catenin	NM_001431665.1	F:GCAGCGACTAAGCAGGAAGG R:TCACCAGCACGAAGGACAGT	197
GAPDH	NM_017008.4	F: ATGACTCTACCCACGGCAAG R: TGGGTTTCCCGTTGATGACC	75

### 2.8 Analysis of key proteins in the NAV2-Wnt3a/β-catenin signaling pathway in rat joints

Joint tissues were harvested from rats in each experimental group. Exactly 0.1 g of tissue was weighed, cryogenically pulverized under liquid nitrogen, and homogenized in 1.0 mL of lysis buffer (Catalog No. 20220415, Solarbio) supplemented with 10 μL of protease inhibitor cocktail (Catalog No. A8260, Solarbio) and 10 μL of phenylmethylsulfonyl fluoride (PMSF, Catalog No. P0100, Solarbio). Following thorough vortexing, the homogenate was incubated on ice for 30 minutes and centrifuged at 14,000 × g for 5 minutes at 4°C. The supernatant containing solubilized proteins was collected and stored at -80°C until analysis. Protein concentration was determined using a BCA Protein Assay Kit (Catalog No. PC0020, Solarbio). For sodium dodecyl sulfate-polyacrylamide gel electrophoresis (SDS-PAGE), 50.0 μg of protein from each experimental group was loaded onto separate lanes. Proteins were resolved via SDS-PAGE using a two-step electrophoresis protocol: 80 V for 20 minutes on a 10% resolving gel followed by 120 V for 60 minutes on a 5% stacking gel. Proteins were then transferred to a nitrocellulose (NC) membrane (Catalog No. AR0135-02, Boster Bio. Technol. Co. Ltd., Wuhan, China) via constant-current wet transfer at 200 mA for 60 minutes. The membrane was blocked with 5% non-fat milk powder under gentle agitation at room temperature for 2 hours. Primary antibody incubations were performed overnight at 4°C using the following reagents: Rabbit Anti-NAV2 antibody (135 kDa, 1:500 dilution; Catalog No. bs-11892R, BIOSS Co. Ltd., Beijing, China), Mouse Anti-Wnt3a Antibody (40 kDa, 1:1000 dilution; Catalog No. M006228, Abmart Biomedicine Co., Ltd., Shanghai, China), Mouse Anti-β-Catenin (44C6) mAb (92 kDa, 1:1000 dilution; Catalog No. M24002F, Abmart), and Rabbit Anti-GAPDH antibody (38 kDa, 1:5000 dilution; Catalog No. bsm-33033M, BIOSS). Subsequently, secondary antibodies—Goat Anti-Rabbit IgG Antibody (1:15,000 dilution; Catalog No. SE134, Solarbio) and Goat Anti-Mouse IgM Antibody (1:15,000 dilution; Catalog No. K0055G, Solarbio)—were applied, and the membrane was incubated at room temperature for 1 hour. The membrane was then extensively washed with TBST. An ultra-sensitive ECL-ready-to-use luminescent substrate (Catalog No. AR1172, Boster) was added for color development. Protein bands were visualized and imaged using the ChemiDoc™ Imaging System (Universal Hood II, Bio-Rad Laboratories, Inc., Singapore), and gray value quantification was performed with ImageJ software (Version 1.53, National Institutes of Health, Bethesda, MD, USA).

### 2.9 Localization analysis of the core proteins in the NAV2-Wnt3a/β-catenin pathway in the joints of rats

The previously dried rat joint sections were retrieved. Sections were subjected to standard dewaxing and rehydration procedures using xylene and graded ethanol series, followed by washing with phosphate-buffered saline (PBS). Antigen retrieval was performed by incubating sections in Tris-EDTA buffer (Catalog No. AR0023-1, Boster) at 95°C for 10 minutes. Following thermal treatment, sections were allowed to cool to room temperature and rinsed again with PBS. Subsequently, sections were treated with endogenous peroxidase-blocking solution for 20 minutes at room temperature, followed by three PBS washes. Nonspecific binding was blocked by applying a protein-blocking solution for 30 minutes at room temperature. Following removal of the blocking solution, Rabbit Anti-NAV2 antibody (diluted 1:1000 in PBS), Mouse Anti-Wnt3a Antibody (diluted 1:100 in PBS), and Mouse Anti-Beta-Catenin (44C6) mAb (diluted 1:100 in PBS) were added dropwise, respectively. Sections were incubated overnight at 4°C and subjected to three PBS washes. Following this, sections were incubated with secondary antibody at 37°C for 30 minutes, followed by PBS washing. Streptavidin-peroxidase conjugate was then applied for 30 minutes at 37°C, with subsequent PBS rinsing. Color development was performed using DAB chromogenic solution (Catalog No. DA1010, Solarbio) for 8 minutes, after which sections were rinsed with tap water. Hematoxylin counterstaining was selectively applied based on protein expression localization. Positive expression areas were quantified using ImageJ software (Version 1.53, National Institutes of Health).

### 2.10 Statistical analysis

Data analysis and visualization were conducted using GraphPad Prism 8.0.2 (GraphPad Software, Inc., La Jolla, CA, USA). All measurements were presented as mean ± standard deviation (x± SD). Statistical analysis employed one-way analysis of variance (ANOVA), with independent t-tests performed for intergroup pairwise comparisons. A p-value < 0.05 was considered statistically significant.

## 3 Results

### 3.1 The effect of MAT on the parameters of rats in different experimental groups

**Figure 1:**
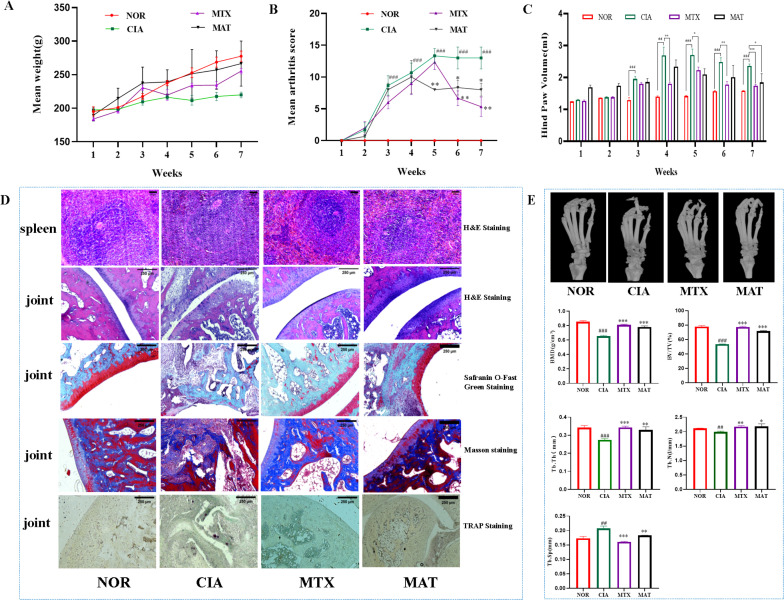
The effect of MAT on the parameters of rats in different experimental groups. **(A)** The body weights of rats in different experimental groups showed no significant difference. Compared with the CIA group, the arthritis score **(B)** and hind paw volume **(C)** in the MAT group were decreased (all *p*<0.05). **(D)** Compared with the CIA group, the MAT group showed a decrease in lymph node volume, the joint surface of rats became smooth and complete, and the infiltration of inflammatory cells was reduced (HE staining). The degree of cartilage damage in the MAT group rats was relieved, the joint surface became flat (Safranin O-fast green staining), the degree of synovial cell proliferation alleviated, the degree of bone erosion improved (Masson staining), and the number of osteoclasts decreased to varying degrees (TRAP staining). **(E)** Compared with the CIA group, the MAT group showed improvement in ankle joint bone destruction in rats. The BMD, BV/TV, Tb.Th, and Tb. N increased significantly (*p*<0.001, *p*<0.001, *p*<0.01, *p*<0.05, respectively), while Tb. Sp decreased significantly (*p*<0.01). Compared with the NOR group, ^#^ *p*<0.05, ^##^ *p*<0.01, ^###^ *p*<0.001, respectively; Compared with the CIA group, * *p*<0.05, **p<0.01, *** *p*<0.001 respectively. The annotations in the following figure are consistent with those in this figure.

Body weights across experimental rat groups demonstrated a gradual increase (**Figure 1A**), though intergroup differences remained nonsignificant. Compared to the NOR group (**Figures 1B, C**), both arthritis scores and toe volume were significantly elevated in the CIA group (p<0.01). Macroscopic examination revealed ankle joint stiffness, swelling, and deformity in CIA rats. In contrast to the CIA group, the MAT group exhibited statistically significant reductions both in arthritis scores and toe volume by the seventh week (p<0.05), suggesting that MAT was capable of alleviating the erythema and swelling of ankle joints and effectively relieving the arthritic symptoms in CIA rats. Concurrently, the MTX group exhibited a comparable ameliorative effect.

Pathological examination results of the spleen and joint tissues from rats in each experimental group are presented in **Figure 1D**. In the NOR group, the size and quantity of splenic lymphoid nodules were within the normal range, with no observable abnormalities. Conversely, in the CIA group, significant enlargement and fusion of the splenic lymphoid white pulp and primary germinal centers were evident. This was accompanied by a marked increase in the quantity of splenic lymphoid nodules. In both the MTX and MAT groups, the volume of lymphoid nodules decreased, and the inflammatory response was mitigated. Regarding joint tissues, the articular surface of rats in the NOR group was smooth and intact, with synovial cells covering the surface and cartilage remaining undamaged. In contrast, CIA group rats displayed a narrowed joint space with inflammatory cell infiltration on the articular surface. Severe articular cartilage damage was evident, resulting in significant chondrocyte loss. Synovial hyperplasia occurred, disrupting the joint structure. TRAP staining identified numerous positively stained osteoclasts in the joint tissues of CIA rats. In contrast to the CIA group, HE staining of the MAT group showed improved joint space architecture and diminished inflammatory cell infiltration. Safranin O-fast green staining indicated that the degree of cartilage damage in MAT-treated rats was alleviated, and the articular surface became smoother. Masson staining showed decreased synovial cell hyperplasia, mitigated bone erosion, and significant improvement in the joint space. TRAP staining further demonstrated varying degrees of reduction in osteoclast numbers in the MAT group. These findings suggest that MAT can inhibit joint inflammation, suppress synovial hyperplasia, reduce bone damage and erosion, and promote cartilage repair in CIA rats.

Micro-CT imaging of ankle joints from rats in each experimental group is shown in **Figure 1E**. The articular surface of the ankle joints in the NOR group was flat and smooth, devoid of any noticeable defects or abrasions. In stark contrast, the ankle joints of rats in the CIA group exhibited severe bone erosion, with honeycomb-like erosion spots manifesting on the articular surface. Quantitative analysis of BMD and trabecular bone indices of the ankle joints revealed that, when compared with the CIA group, BMD significantly increased in both the MAT and MTX groups (p<0.001). Notably, the MAT group exhibited significant increases in BV/TV, Tb.Th, and Tb.N (p<0.001, p<0.01, and p<0.05, respectively), accompanied by a significant reduction in trabecular separation (Tb.Sp, p<0.01). These results indicate that MAT promotes trabecular bone formation and mitigates bone destruction in CIA rats.

### 3.2 The impact of MAT on inflammatory cytokines in the serum of rats across experimental groups

**Figure 2:**
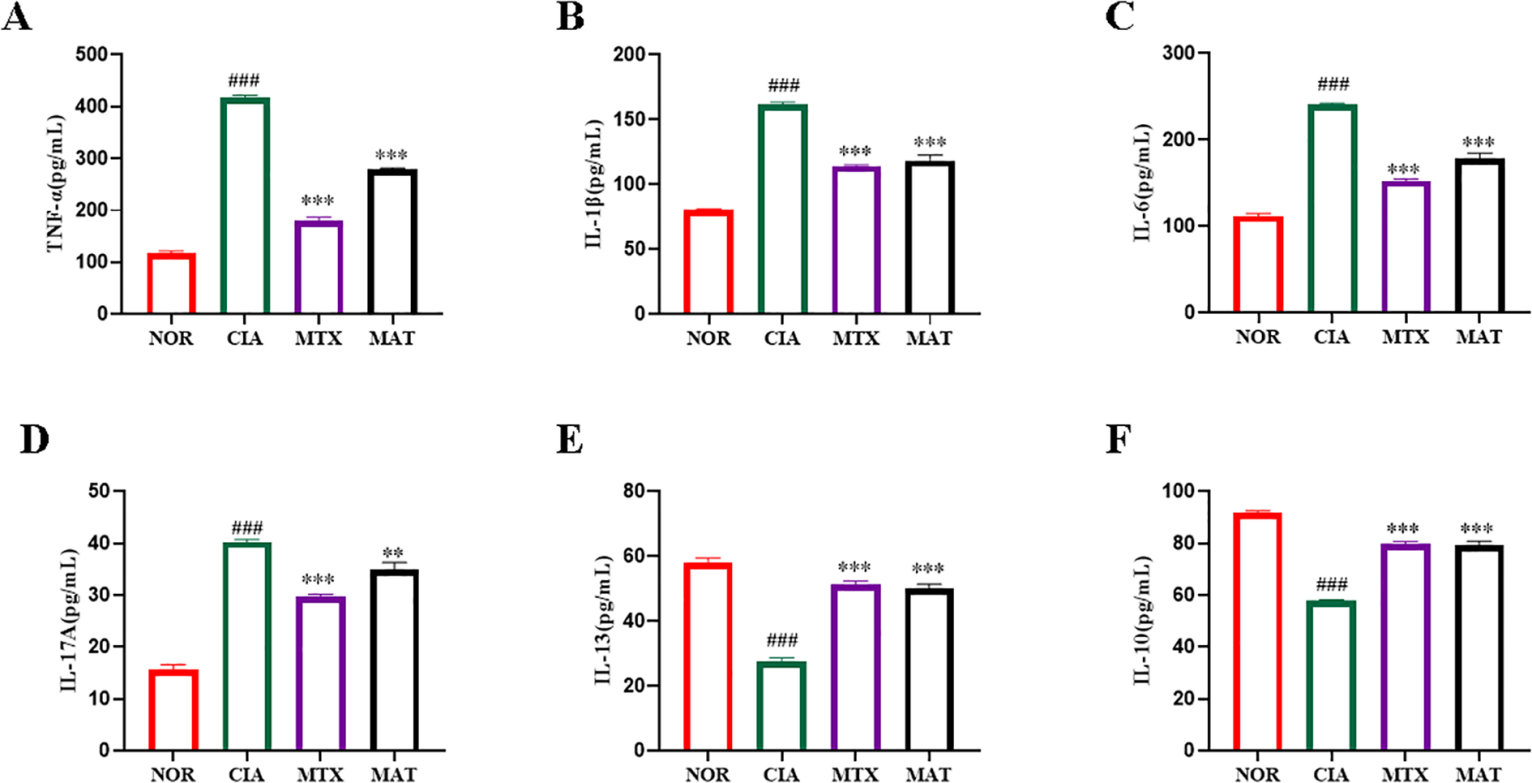
The effect of MAT on inflammatory factors in the serum of rats in different experimental groups. Compared with the CIA group, the MAT group showed a significant decrease in serum pro-inflammatory cytokines TNF-α **(A)**, IL-1β **(B)**, IL-6 **(C)**, and IL-17A **(D)** (*p*<0.001,*p*<0.001,*p*<0.001,*p*<0.01 respectively), while anti-inflammatory cytokines IL-10 **(E)** and IL-13 increased significantly **(F)** (all *p*<0.001). **p<0.01, ***p<0.001, ###*p*<0.001.

As shown in **Figure 2**, serum inflammatory cytokine profiling across experimental groups revealed significant reductions in pro-inflammatory cytokines TNF-α, IL-1β, IL-6, and IL-17A in both the MAT and MTX groups compared to the CIA group (p<0.001, p<0.001, p<0.001, p<0.01, respectively). Conversely, significant increases in anti-inflammatory cytokines IL-10 and IL-13 were observed (p<0.001 for both). These results strongly indicate that MAT potently reduces serum levels of pro-inflammatory cytokines TNF-α, IL-1β, IL-6, and IL-17A while significantly elevating anti-inflammatory cytokines IL-10 and IL-13 in CIA rats.

### 3.3 Detection of safety-related indicators of MAT

**Figure 3:**
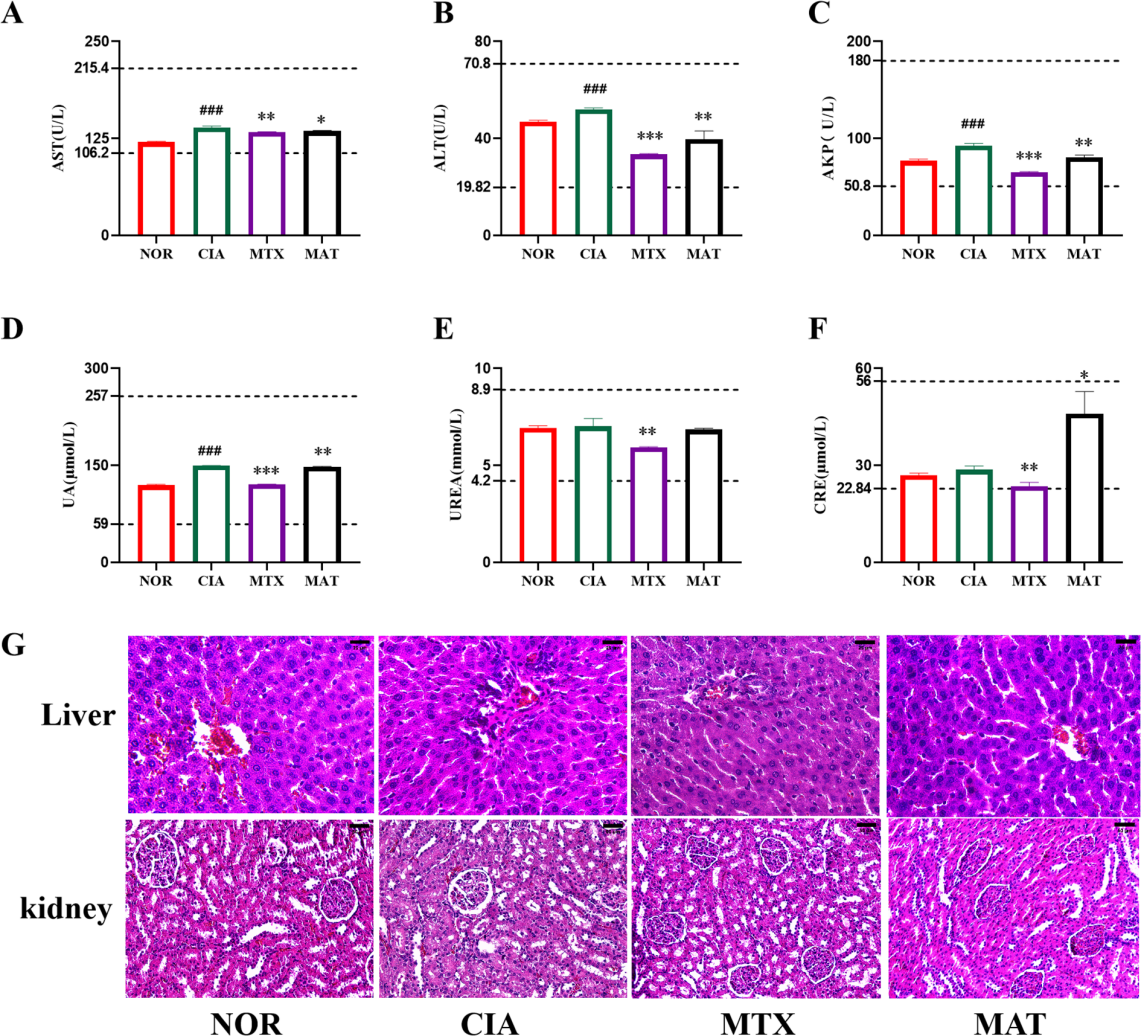
Detection of safety-related indicators of MAT. **(A-F)** represent that AST, ALT, AKP, UA, urea, and CRE, respectively, are within the normal range. The two dashed lines above and below represent the upper and lower limits of normal values. **(G)** The pathological examination results of liver and kidney tissues showed no abnormal pathological changes. *p<0.05, **p<0.01, ***p<0.001, ##*p*<0.001.

As illustrated in **Figures 3A–F**, the detection results of serum biochemical indices related to liver and kidney functions in rats from different experimental groups demonstrated that the levels of AST, AKP, ALT, UREA, UA, and CRE all remained within the normal range. Compared to the CIA group, the MAT group showed a higher CRE level, with statistically significant differences. This differs from the trend of other liver and kidney function indicators, which show either a decrease or no change compared to the CIA group, although the CRE values remained within the normal range. Further research or extended observation periods are necessary to confirm the renal safety of MAT.

Pathological analyses of liver and kidney tissues, as illustrated in **Figure 3G**, revealed that hepatocytes in each group of rats were orderly arranged with an intact structure. The hepatic cords were arranged normally, and the hepatic lobules exhibited a complete morphology, lacking nuclear division, lymphocyte exudation, abnormal necrosis, hyperplasia, or infiltration of inflammatory cells. In renal tissues, renal tubular epithelial cells across all groups were orderly arranged, glomeruli exhibited normal volume and architecture, and no abnormal hyperplasia or inflammatory cell infiltration was detected. These findings strongly suggest that MAT exhibits excellent safety, causing no harm to the liver and kidneys of rats, both serologically and pathologically, thereby indicating its high level of safety.

We will delete the figure and replace it with the following figure.

### 3.4 The impact of MAT on mRNA and protein expression of core genes in the NAV2-Wnt3a/β-catenin pathway in rat joint tissues across experimental groups

**Figure 4:**
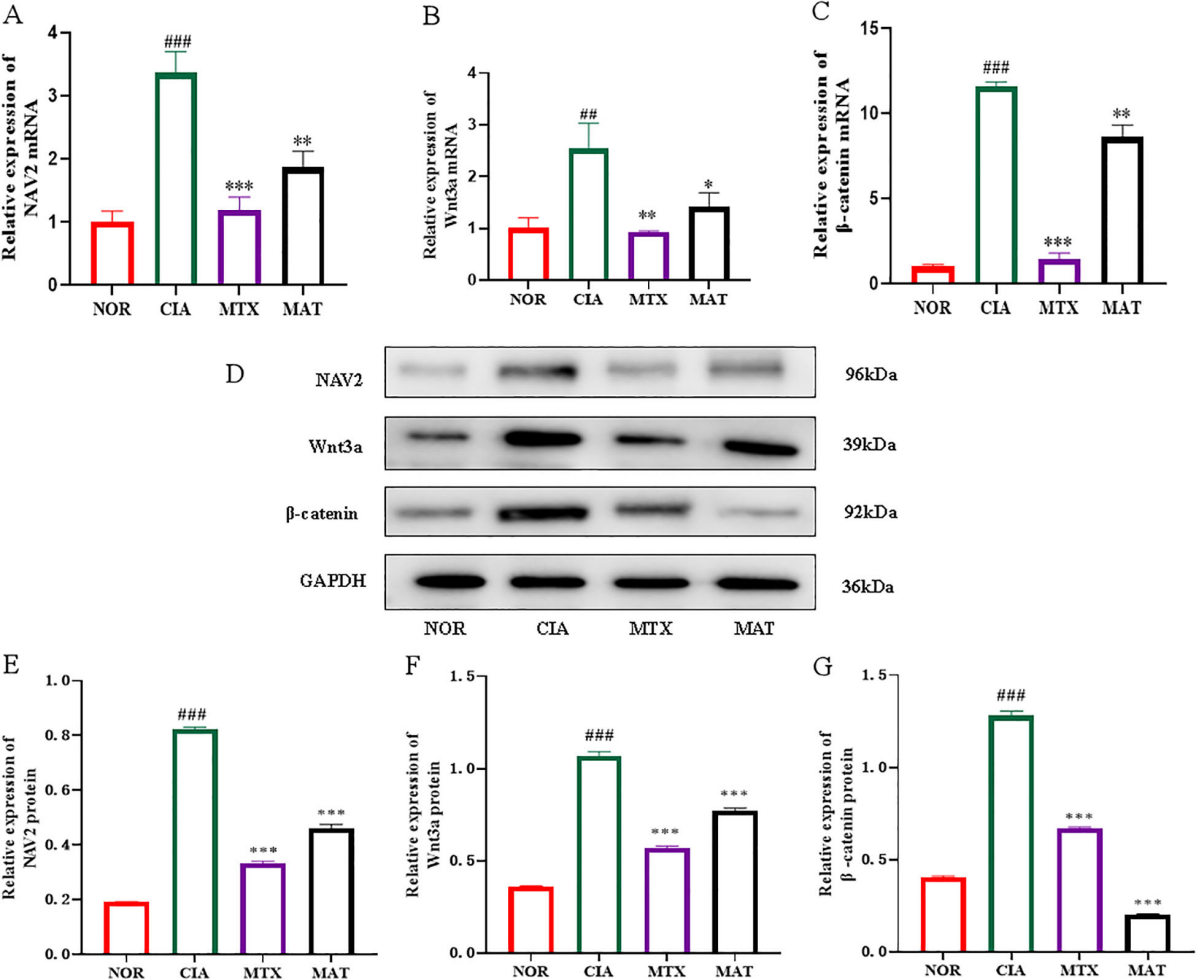
The effect of MAT on the mRNA and protein levels of the core genes in the NAV2-Wnt3a/β-catenin pathway of the joints of rats in different experimental groups. The relative expression of NAV2, Wnt3a, and β-catenin mRNA in the joints of rats in the CIA group was overexpressed. Compared with the CIA group, the relative expression of NAV2 **(A)**, Wnt3a **(B)**, and β-catenin **(C)** decreased in the MAT-treated group(*p*<0.01, *p*<0.05, *p*<0.01, respectively). Western blotting was used to analyze NAV2, Wnt3a, and β-catenin protein expression levels in different experimental groups **(D)**. Compared with the CIA group, the NAV2 **(E)**, Wnt3a **(F)**, and β-catenin protein **(G)** decreased in the MAT-treated group(All *p*<0.001). **p<0.01, ***p<0.001, ###*p*<0.001.

The qRT-PCR results, as depicted in **Figures 4A–C**, showed that compared to the NOR group, expression of NAV2, Wnt3a, and β-catenin in CIA rat joints was significantly upregulated (p<0.001, p<0.01, and p<0.001, respectively). Conversely, in the MAT-treated group, expression of Wnt3a, β-catenin, and NAV2 was significantly downregulated relative to the CIA group (p<0.01, p<0.05, and p<0.01, respectively), with a comparable trend observed in the MTX group. Western blotting was used to assess protein expression of NAV2, Wnt3a, and β-catenin across experimental groups (**Figure 4D**). The protein level detection results, illustrated in **Figures 4E-G**, showed that expression of NAV2, Wnt3a, and β-catenin in CIA rat joints was increased relative to the NOR group (p<0.001). Conversely, expression of these genes in both the MTX and MAT groups was significantly decreased compared to the CIA group (p<0.001 for all). Collectively, these results strongly suggest that the NAV2-Wnt3a/β-catenin axis is highly active in RA and contributes to the pathological process of this disease. MAT, on the other hand, can inhibit the function of the NAV2-Wnt3a/β-catenin axis at both the transcriptome and protein levels, thereby exerting a critical role in the management of RA.

### 3.5 Analysis of the localization of core proteins in the NAV2-Wnt3a-β-catenin pathway in the joints of rats in different experimental groups

**Figure 5:**
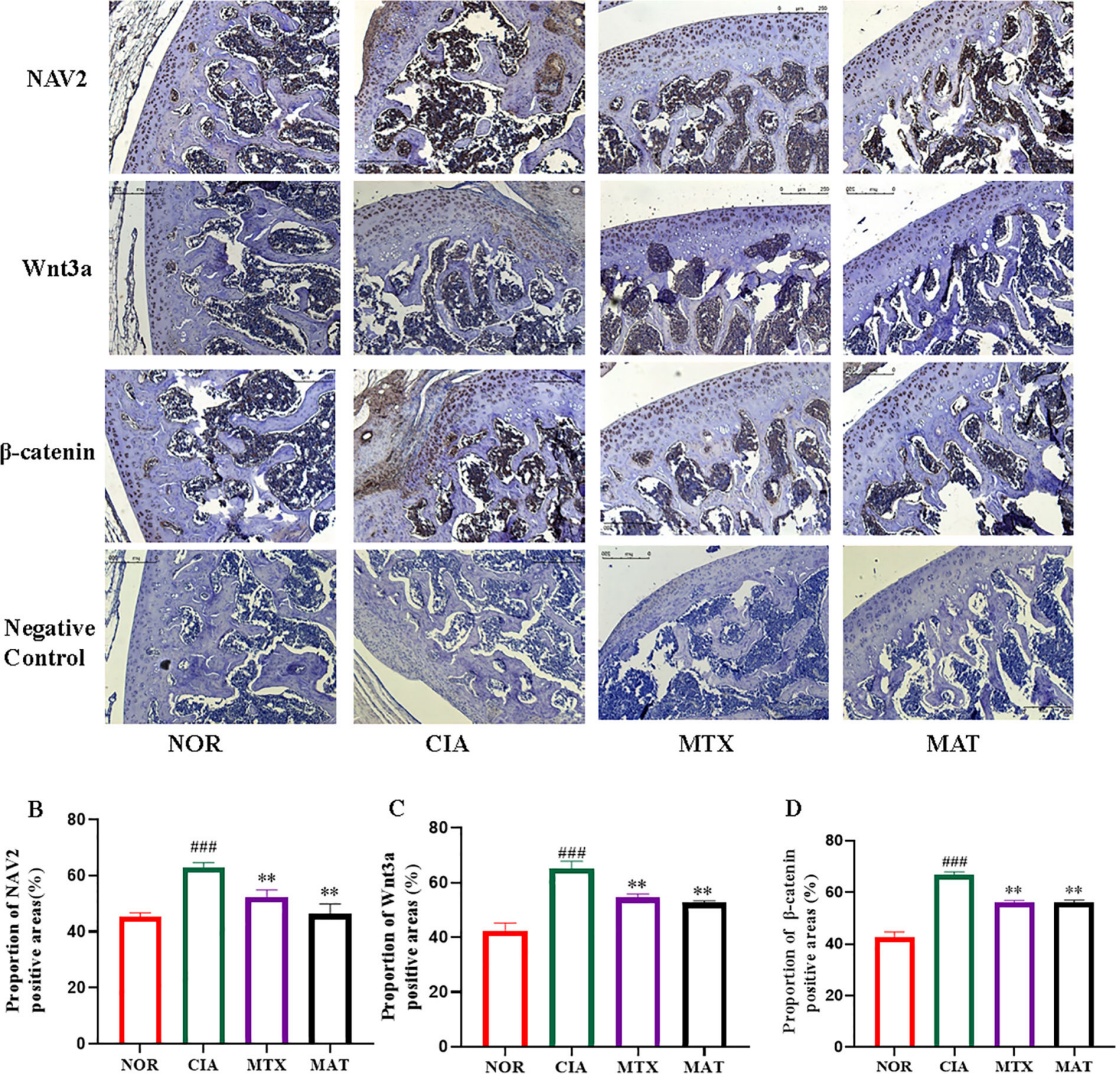
Analysis of the localization of core proteins in the NAV2-Wnt3a-β-catenin pathway in the joints of rats in different experimental groups. **(A)** NAV2 is highly expressed in synovial tissue, while Wnt3a and β-catenin are highly expressed in cartilage and synovial tissue. **(B-D)** The grayscale analysis results showed that compared with the CIA group, the expression of NAV2, Wnt3a, and β-catenin in the synovial and joint tissues of the MAT group rats was significantly reduced (all *p*<0.01). **p<0.01, ###*p*<0.001.

As illustrated in **Figure 5A**, the immunohistochemistry results demonstrated that when juxtaposed with the NOR group, NAV2, Wnt3a, and β-catenin were detected in synovial and cartilage tissues of CIA rat joints. NAV2 manifested abundant expression predominantly in synovial tissues, whereas both Wnt3a and β-catenin exhibited high-level expression in both cartilage and synovial tissues. In contrast to the CIA group, expression of NAV2, Wnt3a, and β-catenin was significantly reduced in synovial and cartilage tissues of MTX- and MAT-treated rat joints. Grayscale analysis (**Figure 5B**) showed that compared to the NOR group, expression of these genes in CIA rat synovial and joint tissues was markedly increased with highly significant differences (p<0.001 for all). Conversely, expression in the MAT group was significantly decreased relative to the CIA group with highly significant differences (p<0.01 for all).

## 4 Discussion

MAT, the principal bioactive monomer derived from the traditional Chinese herb Sophora flavescens, exerts a broad spectrum of pharmacological effects, encompassing anti-tumor, antiviral, anti-fibrotic, anti-inflammatory, and immunomodulatory activities (13, 16–19). Previous studies have shown that MAT alleviates bone destruction in CIA rats by upregulating osteoprotegerin (OPG) expression, downregulating receptor activator of nuclear factor kappa-B ligand (RANKL) expression, and increasing the OPG/RANKL ratio, thereby promoting bone damage repair (20). Findings from the present study revealed that MAT significantly decreased the toe volume and arthritis index (AI) score in CIA rats, consistent with previous research outcomes (21). Micro-CT imaging visually demonstrated that MAT improved bone density parameters, including BMD, BV/TV, Tb.Sp, and Tb.Th. Moreover, the joint pathological analysis indicated that MAT could reduce the number of osteoclasts within joints, effectively alleviating bone destruction. In terms of safety evaluation, MAT exhibited no signs of toxicity in both liver and kidney serological and pathological assessments, suggesting its favorable safety profile.

To further elucidate the specific mechanism underlying MAT’s therapeutic effect on RA, this study demonstrated that MAT could significantly decrease the levels of pro-inflammatory cytokines, including IL-1β, TNF-α, IL-17A, and IL-6, in the serum of CIA rats. Concurrently, MAT significantly elevated the levels of anti-inflammatory cytokines IL-10 and IL-13, consistent with previous study findings. The potential mechanism might involve MAT’s ability to exert immunomodulatory effects by rectifying the Th1/Th2 imbalance and reducing the proportion of Th17 cells, thereby regulating the cytokine profile (13, 22). This research also uncovered the therapeutic efficacy of MAT in RA at the pathological level. In the MAT-treated CIA rat group, significant reductions were observed in the primary germinal centers and lymphoid nodules in splenic pathology. Regarding ankle joint pathology, both the number of synovial inflammatory cells and the degree of synovial cell hyperplasia were decreased. Previous studies have reported that MAT can inhibit inflammation by suppressing the formation of fibroblast-like synoviocytes and pannus (21, 23). Collectively, these results suggest that MAT exerts its therapeutic effects through multiple pharmacological mechanisms, including anti-inflammation, inhibition of bone destruction, and suppression of synovial hyperplasia. Moreover, its favorable safety profile makes MAT a promising novel candidate among traditional Chinese medicine monomers for the treatment of RA.

The Wnt3a/β-catenin signaling pathway represents a fundamental regulatory cascade that intricately orchestrates diverse biological processes, including cell proliferation, migration, tissue homeostasis, regeneration, and embryonic development (24). Accumulating evidence indicates that dysregulation of this pathway contributes to the pathogenesis of a wide array of diseases, encompassing cancers, vascular disorders, and autoimmune conditions (25–27). Notably, RA has been strongly associated with the aberrant activation of the Wnt3a/β-catenin signaling pathway, which actively participates in multiple pathological mechanisms, such as the maintenance, differentiation, proliferation, and self-renewal of synovial cells (25, 28). Consequently, the Wnt3a/β-catenin signaling pathway has been established as a novel therapeutic target for RA (29–31). Previously published studies have firmly established that numerous botanical-derived pharmaceuticals can effectively impede the proliferation, invasion, and migration of synovial fibroblast-like cells in RA through inhibition of the Wnt3a/β-catenin signaling pathway (32–35). NAV2, as a member of the neuron navigator (NAV) protein family, has been implicated in tumor invasion, as well as lymph node and distant metastasis (8). Studies have demonstrated significantly upregulated NAV2 expression in RA patients. Furthermore, TNF-α can markedly augment the NAV2 level, thereby promoting the proliferation and invasion of synovial fibroblast-like cells and subsequently inducing activation of the Wnt3a/β-catenin signaling pathway. Conversely, experimental evidence has demonstrated that inhibiting the expression of NAV2 can substantially mitigate the severity of RA and decelerate disease progression (14). Collectively, these observations strongly suggest that NAV2 may serve as a novel molecular mediator in the treatment of rheumatoid arthritis (36).

This study demonstrated that NAV2, Wnt3a, and β-catenin were detected in the synovial and cartilage tissues of joints, as well as the spleen tissues of CIA rats. Specifically, NAV2 predominantly showed abundant expression in synovial tissues, whereas both Wnt3a and β-catenin exhibited high-level expression in both cartilage and synovial tissues, consistent with previous research (14). Compared to the CIA group, both qualitative and quantitative analyses revealed reduced expression of NAV2, Wnt3a, and β-catenin in synovial and cartilage tissues of MAT-treated CIA rats. Analyses of transcriptome and protein expression in joint tissues revealed a significant reduction in the levels of NAV2, Wnt3a, and β-catenin. Collectively, these multi-level and multi-dimensional findings strongly confirm that MAT exerts its therapeutic efficacy on the rheumatoid arthritis animal model through suppression of the NAV2-Wnt3a-β-catenin signaling cascade. Hence, the results of this study may offer novel perspectives on the pharmacological mechanism underlying MAT’s anti-inflammatory effect on RA.

Currently, although there is a wide variety of medications available for the treatment of RA, including traditional disease-modifying antirheumatic drugs (DMARDs), biological DMARDs, and small-molecule DMARDs, adverse events such as infections, tumors, cardiovascular incidents, and thrombosis frequently occur (37–39), Consequently, a substantial number of treatment requirements remain unfulfilled in clinical practice. This study demonstrated that MAT significantly reduced the inflammatory response and ameliorated bone destruction in CIA rats. Additionally, it preliminarily explored the mechanism by which MAT exerts its therapeutic effect on RA, which involves the inhibition of the NAV2-Wnt3a-β-catenin signaling pathway. These findings strongly suggest that MAT holds great promise as a valuable candidate for the clinical development of novel therapeutic strategies for rheumatoid arthritis. Notably, this study represents only a preliminary investigation at the animal level and is associated with several limitations. Future research will focus on further validating its mechanism of action in fibroblast-like synoviocytes and transgenic mouse animal models, aiming to provide a more comprehensive understanding of the therapeutic potential of MAT.

In conclusion, MAT specifically targets and inhibits the NAV2-Wnt3a/β-catenin signaling pathway. By dual-regulating and restoring the balance of the inflammatory cytokine network, it effectively reverses the pathological processes associated with RA. Moreover, MAT exhibits no signs of liver and kidney toxicity, thereby providing a novel candidate molecule and a targeted mechanism for the intervention of autoimmune diseases, including RA, using natural drugs.

## Data Availability

The raw data supporting the conclusions of this article will be made available by the authors, without undue reservation.
